# X-rays only when you want them: optimized pump–probe experiments using pseudo-single-bunch operation

**DOI:** 10.1107/S1600577515001770

**Published:** 2015-04-02

**Authors:** M. P. Hertlein, A. Scholl, A. A. Cordones, J. H. Lee, K. Engelhorn, T. E. Glover, B. Barbrel, C. Sun, C. Steier, G. Portmann, D. S. Robin

**Affiliations:** a Lawrence Berkeley National Laboratory, 1 Cyclotron Road, Berkeley, CA 94720, USA; b Department of Physics, University of California, 366 LeConte Hall, MC 7300, Berkeley, CA 94720, USA

**Keywords:** pump–probe spectroscopy, bunch kicking, pseudo single bunch, X-ray pulse picking, time-resolved X-ray spectroscopy, sample damage, laser/X-ray measurements, X-ray streak camera

## Abstract

Laser pump–X-ray probe experiments require control over the X-ray pulse pattern and timing. Here, the first use of pseudo-single-bunch mode at the Advanced Light Source in picosecond time-resolved X-ray absorption experiments on solutions and solids is reported. In this mode the X-ray repetition rate is fully adjustable from single shot to 500 kHz, allowing it to be matched to typical laser excitation pulse rates. Suppressing undesired X-ray pulses considerably reduces detector noise and improves signal to noise in time-resolved experiments. In addition, dose-induced sample damage is considerably reduced, easing experimental setup and allowing the investigation of less robust samples. Single-shot X-ray exposures of a streak camera detector using a conventional non-gated charge-coupled device (CCD) camera are also demonstrated.

## Abstract 2

The first use of pseudo-single-bunch pulse picking in time-resolved experiments at the Advanced Light Source is reported. The pseudo-single-bunch technique improves signal to noise and drastically reduces dose-induced sample damage.

## 1. Introduction   

Synchrotron X-ray sources are versatile tools for the study of electronic and structural properties of materials and are employed in a wide range of applications stretching from biology and chemistry to physics and materials sciences. Time-gated experiments use the pulsed nature of synchrotron X-rays, generated by the short electron bunches in storage rings. Such stroboscopic pump–probe or time-of-flight experiments often demand X-ray pulses with high peak power, but also require low to medium repetition rates, from single shot up to 1 MHz, well below the natural repetition rate of synchrotron sources. The temporal spacing between X-ray pulses is typically of the order of just a few nanoseconds.

Pseudo-single-bunch kick-and-cancel (PSB-KAC) is a new operational mode at the Advanced Light Source (ALS) that provides full timing and repetition rate control for single X-ray pulses while being fully transparent to other users of synchrotron radiation (Sun *et al.*, 2012 ▶, 2013 ▶). In this paper we discuss the first use of this new operational mode in measurements of the picosecond dynamics of spin crossover molecules, and in single-shot exposures of an X-ray spectrograph-coupled streak camera. We achieve a considerable reduction in the background and an improvement in signal to noise. PSB-KAC also enables usage of low-noise charge integrating detectors in time-resolved experiments, for example slow-scan CCD cameras or large-area photodiodes.

We begin with a very brief overview of PSB-KAC and of a typical experimental setup utilizing this mode, followed by the discussion of the experimental results. In the final section, we discuss details of the PSB-KAC data collection setup and the measured noise suppression at beamline 6.0.2.

## 2. The ‘pseudo-single-bunch kick-and-cancel’ technique   

**Figure 1:**
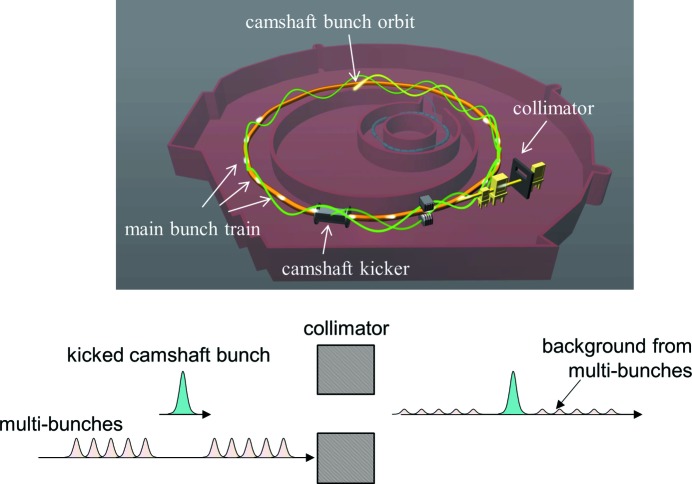
Top: orbit of the multi-bunch train and the kicked camshaft bunch in the storage ring. The kicker magnet periodically moves the camshaft into a different orbit. Bottom: the X-ray beam emitted from the multi-bunches is blocked by a horizontal slit, also called the vertical beam-defining aperture, while X-rays generated by the kicked camshaft pulse are displaced vertically and pass through.

The idea behind pseudo-single-bunch (PSB) operation is to use a high-repetition-rate (MHz) short-pulse (<100 ns) kicker to vertically displace a single electron bunch, the so-called camshaft bunch, relative to the multi-bunch train (Sun *et al.*, 2012 ▶, 2013 ▶). By blocking the light from the multi-bunch train at a collimator in the beamline, only light from the camshaft bunch reaches the experiment (Fig. 1 ▶). Periodically exciting the kicker sends the camshaft bunch on an oscillating trajectory, while the multi-bunch train is not affected. By choosing the right kicker pulse pattern and storage ring lattice, the camshaft bunch can first be displaced to a different orbit and then kicked back to its original orbit within a few turns. This kick-and-cancel process can be repeated at user-selectable intervals, thus creating single X-ray pulses with adjustable repetition rate from single shot to 0.5 MHz.

Functionally similar pulse picking schemes have been developed at other synchrotron sources. Holldack *et al.* (2014 ▶) describe a resonant excitation scheme of betatron oscillations of a single storage ring bunch, which produces a horizontally displaced X-ray pulse at fixed MHz frequency. Jiang *et al.* (2014 ▶) propose a technique related to PSB as a means to create two spatially separated bunch trains in a storage ring.

## 3. Setup and data   

ALS beamline 6.0.2 is optimized for stroboscopic experiments using synchronized ultrafast laser pulses at a low repetition rate between single shot and 4 kHz. A combination of optimized X-ray optics, multiple apertures, a fast-spinning mechanical chopper and electronic gating provide an extremely effective suppression of the background signal generated by the multi-bunch train. Using the mechanical chopper paired and synchronized with PSB-KAC mode results in a total background suppression factor of over 200000, permitting low-repetition-rate and single-shot experiments using integrating detectors. While the displaced camshaft pulse from PSB-KAC is accessible at all ALS beamlines, the degree of suppression of the multi-bunch background will depend on the individual beamline layouts.

**Figure 2:**
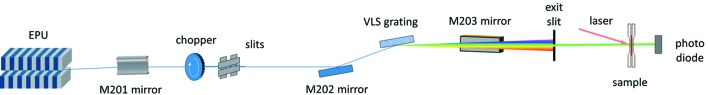
Schematics of the beamline optics at ALS beamline 6.0.2.

Fig. 2 ▶ shows the setup used at beamline 6.0.2 (Heimann *et al.*, 2007 ▶). In spectrometer mode operation, the X-rays generated in the storage ring by the elliptically polarizing undulator (EPU) are first imaged by toroidal mirror M201 to a group of horizontal and vertical entrance slits defining a rectangular entrance aperture. The horizontally oriented adjustable slit serves as both entrance slit to the following X-ray monochromator and PSB pulse selection slit. The vertically oriented slit creates the object for the M203 horizontal refocusing optics. The X-ray chopper, operating at 4 kHz, is installed before the slits in order to lower thermal loading on downstream optics, and reduces the average transmitted beam power by a factor of 30.

The monochromator consists of a spherical mirror (M202) and a variable-line-spaced (VLS) plane grating and disperses and vertically focuses the X-ray beam to the horizontal exit slit near the sample. A final elliptical mirror (M203) provides horizontal focusing of the beam. The X-ray energy transmitted through the slit is adjusted by varying the VLS grating incidence angle. The sample is placed behind the exit slit at the focus of M203, and is excited by a laser pulse that is synchronized with the PSB X-ray pulse. An X-ray sensitive avalanche or large-area photodiode then detects the transmitted, reflected or scattered X-ray intensity.

Alternatively, the beamline can be used in spectrograph mode, where a transmission sample is inserted into the beam in a pink beam vacuum chamber immediately behind the entrance slits. In this mode the beamline exit slit is removed, and the energy-dispersed transmitted X-ray intensity is detected by an area detector or streak camera behind the M203 optics.

### 3.1. Pump–probe measurements on solvated Fe^II^   

The Fe^II^ polypyridyl complex {Fe[tren(py)_3_]}^2+^ is part of a widely studied class of spin crossover complexes that undergo a transition from a low (*S* = 0) to a high spin state (*S* = 2) after photoexcitation (Huse *et al.*, 2010 ▶, 2011 ▶). Of fundamental interest is the coupling between electronic, spin and orbital degrees of freedom and the speed and energetics of the excitation and relaxation processes following an external excitation. The Fe^II^ complex is a prototypical system for ultrafast pump–probe-style studies of electron and chemical dynamics in molecules and has been studied previously using femtosecond and picosecond X-ray absorption spectroscopy. Near-edge X-ray absorption fine-structure spectroscopy (NEXAFS) is an element-specific probe of the electronic structure of a molecule, based on a core electron promotion into empty valence states following the absorption of an X-ray photon. The absorption spectrum explores the symmetry, spin state and density of states of the valence electronic structure. The transient electron and structural dynamics of the molecule can be measured using a laser pump–X-ray probe scheme, measuring the X-ray response following an optical excitation pulse by a visible or infrared femtosecond laser pulse.

Previous studies showed a sub-picosecond spin crossover conversion from a metal-to-ligand charge transfer (MCLT) state to a high spin state after optical excitation. These measurements were carried out at 4 kHz repetition rate using 70 ps X-ray pulses, synchronized to the femtosecond pump laser (Huse *et al.*, 2010 ▶). Further experiments used 200 fs X-ray pulses from the ALS slicing source (Huse *et al.*, 2011 ▶). The sample consisted of a 100 m*M* solution of Fe^II^ molecules in acetonitrile in a 2 µm-thick silicon nitride cell, and was measured in transmission. Since the material in this static cell could not be replenished during the measurement, care had to be taken to minimize the X-ray exposure and limit sample damage during the experiment. Prior to the availability of PSB-KAC this was accomplished by reducing the X-ray flux through detuning the insertion device and closing beamline apertures, which also lowered the otherwise achievable signal-to-noise ratio. This flux reduction was necessary because, even with the rotary chopper blocking 97% of X-ray pulses, 1199 out of 1200 photons reaching the sample were not timed correctly with the exciting laser pulse and thus useless for data collection, yet they still contributed to sample damage and had to be electronically rejected using a fast, gated boxcar integrator.

**Figure 3:**
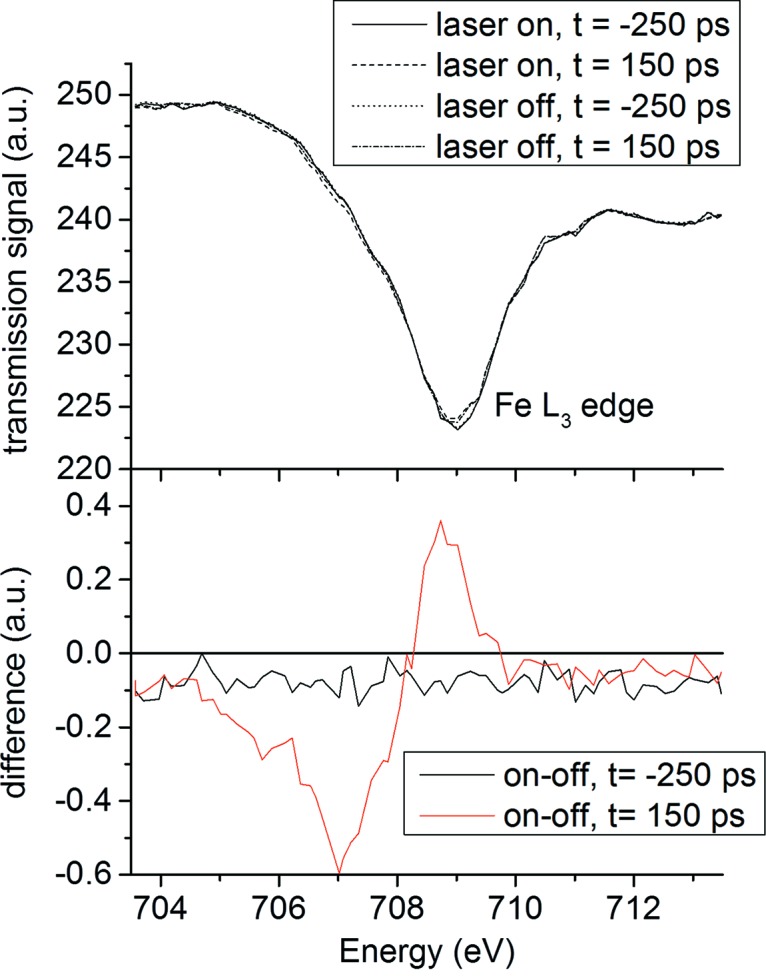
Fe *L*_3_-edge transmission spectra and pump–probe difference of solvated Fe^II^ polypyridyl measured using PSB-KAC (top). Four spectra were acquired (before/after time zero and with laser on/off), showing a small transient change that is better visible in the difference spectra (bottom). Negative delay indicates an X-ray measurement before the laser excites the sample.

Here we report measurements on an identical sample using PSB-KAC, which reduces the repetition rate of the synchrotron pulse train to 4 kHz, exactly matching the frequency of the laser and of the detector readout. Fig. 3 ▶ shows X-ray transmission measurements of the Fe *L*_3_-edge. A reduction factor of 120 in X-ray dose and potential damage was achieved by suppressing the radiation originating from multi-bunches while at the same time increasing the detected signal from a single camshaft pulse by a factor of ten. The latter was possible because no detuning of the beamline was needed to prevent radiation damage.

The transmitted X-ray intensity was measured as a function of photon energy using a gated avalanche photodiode (APD), with data collection synchronized to the PSB X-ray pulse at 4 kHz. The signal was converted using a boxcar analyzer, with its gate timing centered on the PSB camshaft pulse, integrating the transmitted X-ray signal of the reverse-biased APD at 350 MHz bandwidth. The boxcar signal was digitized and then read and accumulated by the data acquisition system. In order to measure the differential change in the X-ray transmission due to laser excitation, the pulsed laser was modulated on and off at 2 kHz, interleaving optically pumped and not-pumped measurements of the state of the sample. The measurements were also repeated at two different time delays between laser pump pulse and X-ray probe pulse; here, negative delay indicates an X-ray measurement before the laser excites the sample. The post time-zero difference spectrum shows a strong differential effect indicating a shifting of spectral weight from 708.7 eV to 707 eV in accordance with previous measurements (Huse *et al.*, 2010 ▶), indicating the optically driven population of the Fe^II^ high spin state. Note that the change in transmitted signal is opposite in sign to the change in absorbance. The 0.5% effect is easily visible at a total integration time of 32 s per energy point. As expected, the differential spectrum acquired at negative delay does not show energy dependence. The small constant offset from zero is due to cross-talk of the gated detection circuit and the laser modulation.

**Figure 4:**
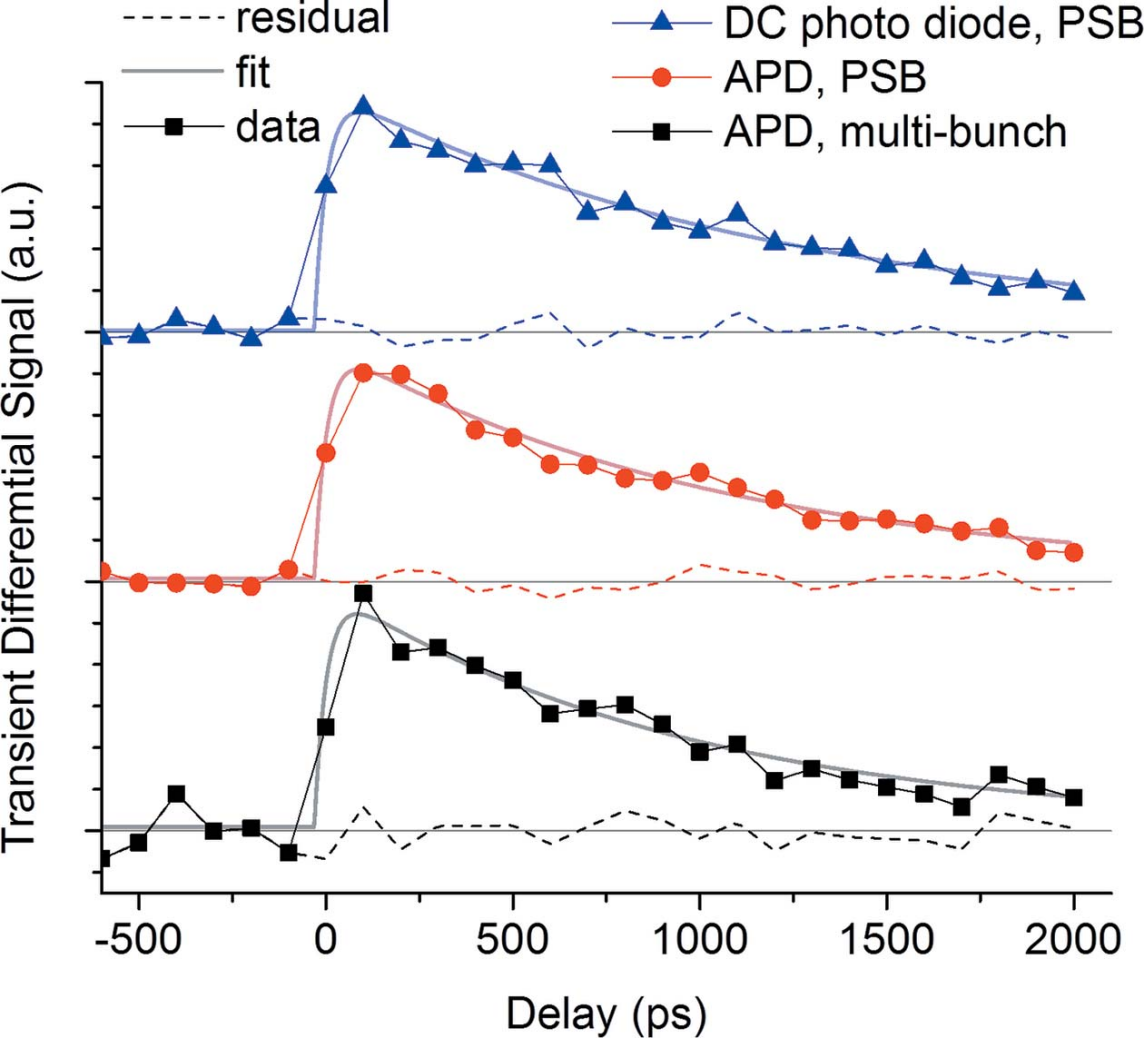
Time dependence of transient spectral changes at 709 eV measured by three different detection modes, with exponential fit and residual. The spectra are stacked vertically for easier comparison. A constant pre-time-zero offset was subtracted and the transient change normalized to unity for easier comparison. All spectra were acquired in sequence and using the same laser power and fluence. Analysis shows that noise is reduced by a factor of up to four using PSB-KAC.

PSB-KAC mode also permits using slow DC-coupled detectors since the X-ray repetition frequency can be easily matched to the repetition rate of the pump pulse. This opens the door to new detection schemes, *e.g.* using two-dimensional CCD cameras, without the need for extremely fast time-gated detectors. We demonstrate this new capability by acquiring transient data using a slow large-area Si photodiode (Opto Diode Corp., model SXUV100). Fig. 4 ▶ shows the time dependence of transient spectral changes at 709 eV measured using three detection schemes, first using traditional time-gated detection using an avalanche photodiode (APD) in multi-bunch mode, second using the same APD in PSB mode, and third using the large-area Si photodiode. All transient spectra were acquired by digitizing the X-ray signal of camshaft pulses at 4 kHz repetition rate. The camshaft pulses were isolated either using boxcar-gated readout of the APD (first and second scheme) or exclusively relying on PSB-KAC pulse picking in the case of the slow photodiode (third scheme). The signal of the X-ray diode was amplified using a current-to-voltage amplifier (SRI570) in high bandwidth mode and digitized at 4 kHz by the data acquisition systems. In all three cases, optically pumped and not-pumped measurements were interleaved at 2 kHz; all measurements used the same total accumulation time of 20 s per point. All three methods show similar transient spectra, starting with a rapid population of the high-spin state, followed by slow relaxation on a nanosecond time scale. The transient spectra were fitted assuming an initial rapid population of the high spin state followed by an exponential decay modeling the relaxation of the molecules back into the low spin state. Good agreement was found. The residual of the fit is a measure of the acquisition noise, which is the sum of statistical noise (shot noise), detector noise, amplifier noise and digitizer noise. Its mean-squared error is an upper limit for the quality of the data, allowing comparison of the three detection methods, as shown is Table 1 ▶.

Using PSB mode in combination with APD detection leads to an approximately four-fold reduction in noise compared with APD detection in multi-bunch mode. X-ray diode detection shows slightly higher noise than APD detection using PSB-KAC, probably because it does not have internal gain like the APD. These data demonstrate that PSB not only provides greater flexibility when using non-gated detection schemes but also leads to improved data in a typical measurement.

### 3.2. Background reduction in time- and energy-resolved streak-camera experiments   

Pump–probe-style experiments at synchrotron sources are limited in time resolution by the length of the X-ray pulse (70 ps at the ALS), and they usually require a process with repeatable dynamics. Streak cameras are used for higher time resolution or for the study of non-repeatable processes that require single-shot data acquisition. A streak camera is a single-shot time-resolved detector that displays the response of a material dispersed in time on an imaging detector. Streak cameras have been used previously at the ALS for the study of magnetic materials and warm dense matter (Opachich *et al.*, 2010 ▶; Feng *et al.*, 2010 ▶; Cho *et al.*, 2011 ▶). In X-ray transmission warm dense matter (WDM) experiments, materials are rapidly heated well above their melting temperature using a high-power femtosecond laser pulse, and their response is measured using NEXAFS at varying times afterwards. Changes in the lattice and electronic structure of the material following laser excitation manifest themselves through changes in the observed absorption edge.

**Figure 5:**
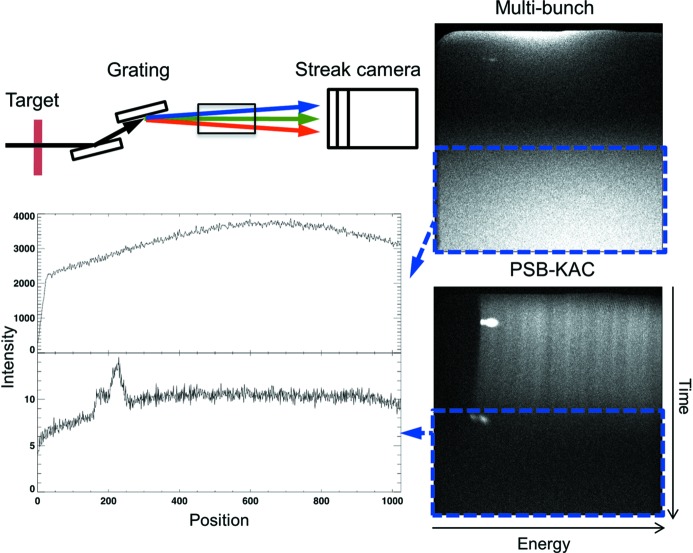
Top left: beamline 6.0.2 in spectrograph mode using an X-ray streak camera detector. Right: energy- and time-dispersed images in multi-bunch and PSB-KAC mode. Bottom left: intensity profiles, vertically averaged over the areas outlined in blue on the right, showing the magnitude of the background signal: top, multi-bunch; bottom, PSB-KAC. Note the difference in vertical scale.

The X-ray streak camera at BL 6.0.2 has a time resolution of 2 ps and uses the beamline in spectrograph mode, dispersing the X-ray signal in time and energy along two coordinates. Fig. 5 ▶ shows a schematic of the measurement setup. A two-dimensional imaging detector acquires single-shot images of the state of the sample using a single camshaft X-ray pulse. The X-ray pulse is dispersed in energy along the horizontal axis and in time along the vertical axes. Since samples are destroyed during the laser exposure, experiments have to be conducted single-shot and are repeated at a low repetition rate of 1 Hz by translating a fresh sample region into the beam after each exposure.

A mechanical shutter with an opening time of 2 ms is placed upstream of the sample behind the X-ray chopper to reduce the amount of X-ray pulses arriving at the sample between laser shots. Even with this shutter, the ratio between correctly timed X-ray signal and background is 1:10000, and damage to the sample in the intense X-ray beam upstream of the monochromator is still significant while the background signal on time-integrating detectors remains high. The streak camera does provide some rejection of pulses not synchronized with the 1 Hz pump laser; however, prior to the availability of PSB-KAC, reducing the high background to a workable level required the addition of an MCP/image intensifier with a gate time of ∼100 ns. This type of detector compromises both sensitivity and spatial resolution when compared with direct electron imaging detectors, which cannot be gated. PSB-KAC allows a direct detection scheme of the streak camera electrons with a back-illuminated CCD camera (Andor), improving the efficiency, temporal and spatial resolution of the experiment. Fig. 5 ▶ shows a direct comparison of the signal and background using conventional multi-bunch mode and PSB-KAC while using the direct imaging detector. PSB-KAC reduces the intensity in the lower half of the image, which is dominated by multi-bunch background, by a factor of ∼300, allowing the correctly timed camshaft X-ray signal and two time fiducials (bright spots on the left) to emerge in the upper half of the image. In addition, X-ray sample damage is greatly reduced because a large fraction of not correctly timed X-ray photons are rejected before the sample. This feature is crucial to the survival of even robust samples (*e.g.* metals); it furthermore permits the study of new materials that are more sensitive to radiation damage.

## 4. PSB-KAC performance at beamline 6.0.2   

The ALS uses two different fill patterns: for about 90% of the year a 500 mA multi-bunch pattern and for about 10% of the year a 35 mA two-bunch pattern. During regular 500 mA operation of the ALS the 500 MHz multi-bunch bunch train consists of 276 consecutive RF buckets (out of 328 available) filled with electrons to a nominal current of about 1.8 mA per bucket. In the remaining 102 ns gap only one single bucket, the camshaft, is filled to a nominal current of about 5 mA.

**Figure 6:**
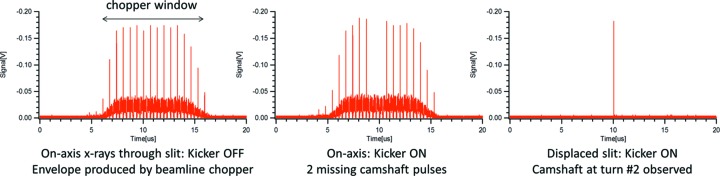
X-ray pulse pattern downstream of the 4 kHz chopper measured by a fast photodiode during regular operation (left), with KAC-PSB active at 4 kHz (center), and with KAC-PSB active and slit displaced to block multi-bunch pulses and transmit one selected camshaft pulse (right).

Fig. 6 ▶ shows X-ray pulses detected at the sample location within a single 4 kHz chopper window, measured by an APD during multi-bunch operation of the ALS. About 12 storage ring round trips with their corresponding camshaft and multi-bunch pulses are transmitted through the chopper per slot opening. During PSB-KAC operation at 4 kHz, the camshaft bunch in the storage ring is displaced for two round trips whose orbit offsets and angles are listed in Table 2 ▶. The camshaft pulses from these two turns are blocked by the on-axis entrance slit. If the slit is displaced, the multi-bunch pulses are steered onto the aperture while one of the camshaft pulses is being transmitted as shown in the third graph.

PSB-KAC operation is also available during two-bunch operation of the ALS during which two equally spaced buckets in the storage ring are filled. Because of a different storage ring tune a more complex kicker pattern is used employing four kicks and five round trips before the camshaft bunch reaches its standard orbit. The kicked bunch is again isolated by rotating M201.

**Figure 7:**
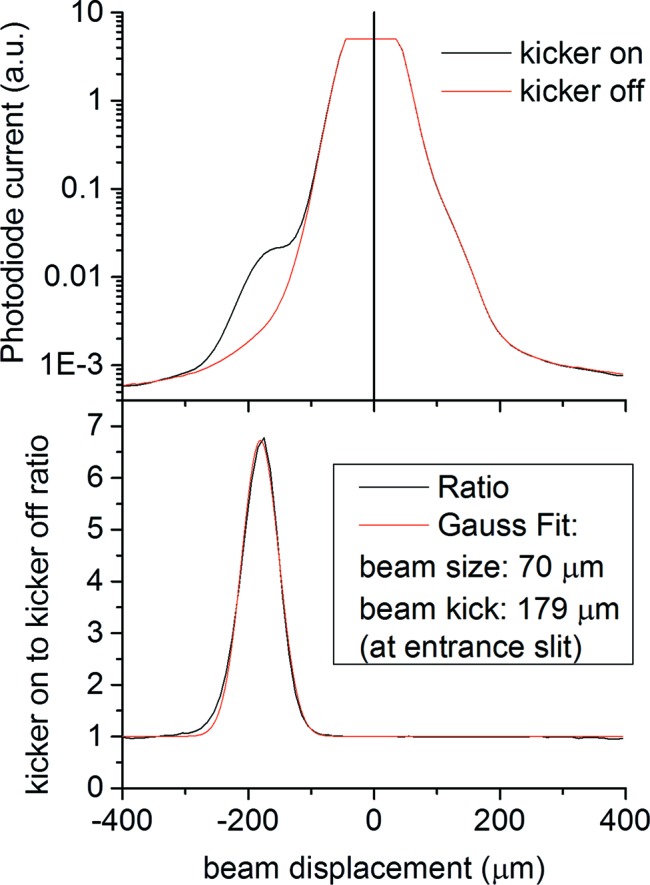
Top: photodiode intensity transmitted through a 10 µm-wide horizontal entrance slit during a M201 roll scan with the 4 kHz PSB kicker turned on/off. The main peak is caused by multi-bunch pulses while the second orbit of the KAC-PSB pulse is visible as a shoulder on the left side of the main peak. Bottom: ratio between the kicker-on and kicker-off intensity.

Ideally, a 100% transmission of the selected camshaft pulse and a total suppression of the multi-bunch background are desired using PSB-KAC. In practice, the suppression of non-modulated pulses is incomplete because of limits in the bunch separation that can be achieved using the existing kicker, the width and profile of the electron bunch, and scattering from and aberrations of the beamline optics. Fig. 7 ▶ shows the vertical X-ray beam profile measured by an integrating photodiode detector as the beam is scanned across the entrance slit (set to 10 µm size) by rolling the M201 mirror. The coordinate of the mirror roll stage can be transformed into a position coordinate at the entrance slit by comparing the beam size measured at the entrance when rotating the M201 mirror and when translating the slit. The figure shows two traces as a function of mirror stage position, one with the PSB kicker turned on, the other with the kicker turned off. The most intense feature in the center originates from not-kicked multi-bunch pulses and is similar in both traces since the intensity reduction from removing kicked camshaft bunches is very small in comparison with the total intensity (about 1 in 1200). The detector intensity is saturated in the center because a high gain was chosen to better detect the small signal from kicked bunches. The vertical beam size is 70 µm (FWHM), a convolution of the 1:1 imaged vertical profile of the electron bunch with the transform function of the first beamline optics. The kicker was operated at 4 kHz, synchronously with the opening window of the X-ray chopper.

Displaced by 179 µm from the multi-bunch peak a shoulder appears in the ‘kicker-on’ curve, which is the signal from the second orbit of the kicked camshaft. This peak sits on a background of scattered X-rays originating from the multi-bunch pulses. The X-rays generated during the first orbit are not visible on the graph since they are displaced less and are overwhelmed by the multi-bunch background. The ratio of pseudo-single-bunch to multi-bunch background is about 6:1 at beamline 6.0.2 and is sensitive to proper focusing of the beamline optics; it also benefits from apertures that reduce off-axis scatter.

A single camshaft bunch carries 1200 times less charge than the total charge-generating X-rays during a chopper opening corresponding to about 12 round trips. The signal from the PSB camshaft pulse is six times more intense than the residual multi-bunch background (Fig. 7 ▶); this means that PSB-KAC suppresses the multi-bunch background by a factor of 7200. The chopper is responsible for an additional suppression of 30:1 resulting in a combined suppression of undesired X-rays of more than 200000:1 at beamline 6.0.2, allowing us to use a 500 MHz X-ray source effectively as a 4 kHz source without fast gating electronics.

PSB-KAC also greatly reduces the X-ray thermal load on optics and potential sample damage downstream of the first aperture of the beamline. The chopper alone lowers the X-ray power from about 300 W to 10 W upstream of the monochromator, and reduces the monochromatic X-ray flux downstream of the monochromator from typically 1 × 10^13^ to 3.3 × 10^11^ photons s^−1^; however, most of these transmitted photons will not be correctly timed in a pump–probe-type experiment. When adding PSB-KAC mode in combination with the chopper on the other hand, the majority of the photons transmitted are correctly timed, and the X-ray power upstream of the monochromator is reduced to 8 mW, with a 2.8 × 10^8^ photons s^−1^ flux downstream of the monochromator. While nearly all types of samples are quickly destroyed by the focused X-ray beam in the pink beam chamber downstream of the entrance slit and chopper (often within milliseconds, at 10 W average power) when using the regular multi-bunch beam, relatively radiation hard samples, *e.g.* metal foils, will survive indefinitely when using PSB-KAC (at ∼8 mW average power). This greatly increases the range of studies that can utilize the spectrograph mode of the beamline. Even without a chopper a 1200:1 reduction of the total power to 0.25 W would be achieved solely by using PSB-KAC. This offers the possibility to dump the majority of the remaining X-ray power in a heavily cooled aperture upstream of the chopper, which allows the use of fast-spinning uncooled choppers for additional background suppression, greatly simplifying beamline design.

**Figure 8:**
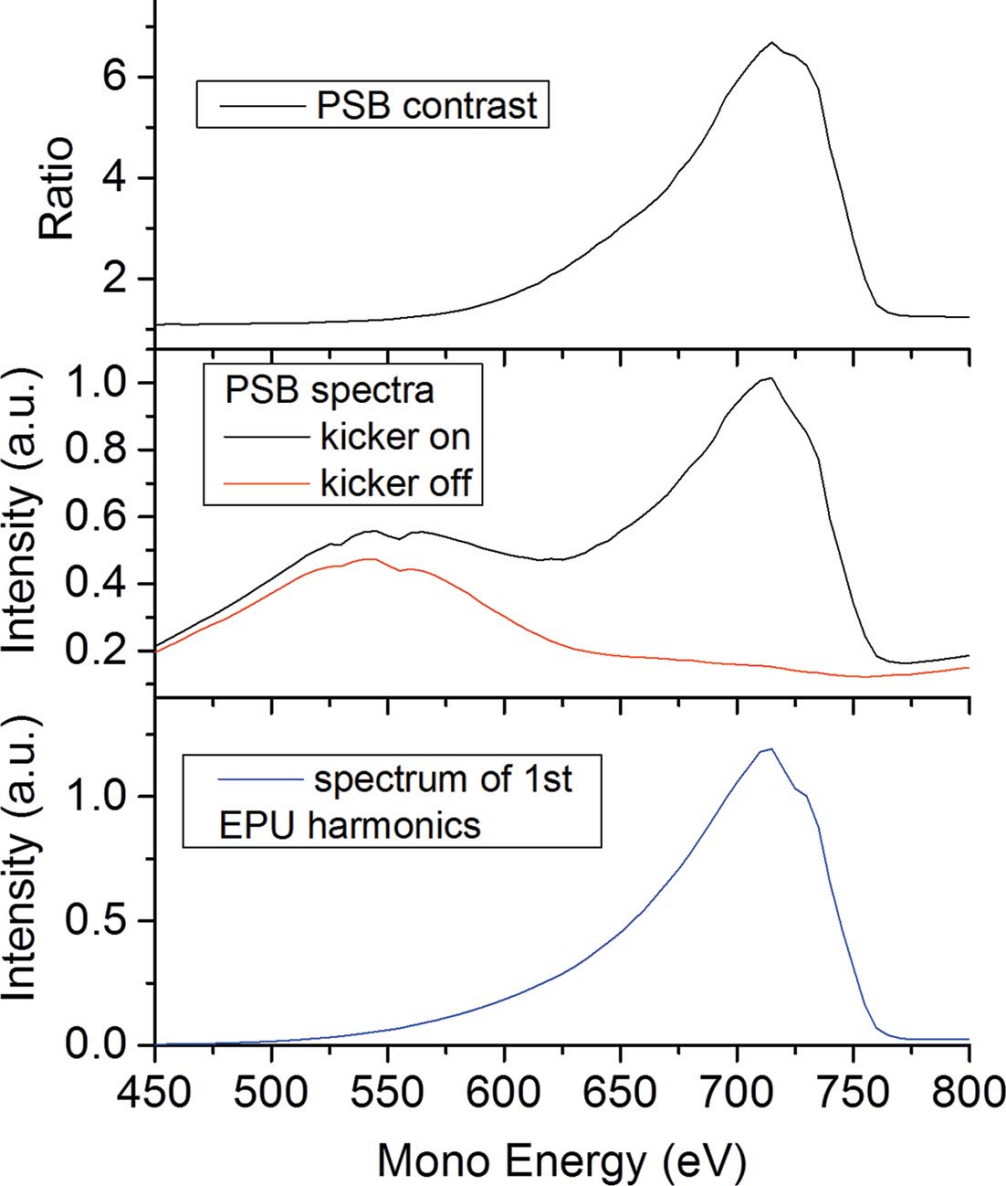
Top: PSB intensity to background ratio across the first undulator harmonics at fixed undulator gap for M201 optimized to deliver PSB radiation. Center: undulator intensity spectra with the kicker turned on/off while using PSB. Bottom: intensity spectrum of the first undulator harmonics at fixed gap with M201 optimized to deliver multi-bunch on-axis.

The residual exposure of the sample to not correctly timed radiation is caused by the multi-bunch background that passes the slit at an off-axis angle. This background is composed of diffuse scatter at mirror M201, which has the same energy dependence as the multi-bunch beam, and the off-axis radiation of the insertion device. The off-axis energy spectrum of an undulator is red-shifted, which is expected to result in an energy-dependent PSB signal-to-background ratio. Fig. 8 ▶ shows that the PSB-to-background ratio peaks near the maximum of the undulator harmonics while the lower-energy region is indeed dominated by red-shifted multi-bunch background. These spectra were acquired by scanning the monochromator across the first undulator harmonic while keeping the undulator gap or *K* value constant. M201 was optimized to deliver PSB radiation and block multi-bunch radiation at the entrance slit. The highest suppression of off-axis background coincides in energy with the peak of the on-axis undulator harmonics. Apparently, the multi-bunch background is dominated by off-axis radiation of the insertion device and not by diffuse scatter of the optics. A further reduction in background can be achieved by increasing the spatial separation of the kicked bunch from the multi-bunch train.

## 5. Conclusions   

The PSB-KAC technique removes many restrictions of low-repetition-rate or time-gated X-ray experiments at high-repetition-rate synchrotron sources by providing user-selectable X-ray repetition rates and effective reduction of unwanted X-rays. At kHz frequencies, using PSB-KAC is transparent to other beamlines because changes in signal are small (1%) and are of very short duration (1.2 µs) compared with the repetition rate of the experiment. PSB-KAC enables the use of time-integrating detectors, drastically lowers the X-ray power on optics and samples, and reduces X-ray dose by many orders of magnitude. We have demonstrated the use of PSB-KAC in pump–probe measurements on spin crossover complexes, and in a WDM demonstration experiment using a time-integrating single-shot streak camera, achieving improved signal to noise while reducing X-ray exposure by three orders of magnitude.

## Figures and Tables

**Table 1:** Comparison of noise of three detection schemes; the mean-square residual of the fit is tabulated

	APD multi-bunch	APD PSB-KAC	Photodiode PSB-KAC
Mean-square error	0.0062	0.0017	0.0023

**Table 2:** Orbit offsets and angles of the kicked camshaft bunch at beamline 6.0.2 for two displaced round trips

	Orbit offset (m)	Orbit angle (rad)
First turn orbit	90	98
Second turn orbit	200	24
